# Risk reclassification analysis investigating the added value of fatigue to sickness absence predictions

**DOI:** 10.1007/s00420-015-1032-3

**Published:** 2015-02-22

**Authors:** Corné A. M. Roelen, Ute Bültmann, Johan W. Groothoff, Jos W. R. Twisk, Martijn W. Heymans

**Affiliations:** 1 Department of Epidemiology and Biostatistics, VU University Medical Center, VU University, Amsterdam, The Netherlands; 2 Department of Health Sciences, University Medical Center Groningen, University of Groningen, Groningen, The Netherlands; 3 ArboNed, PO Box 158, 8000 AD Zwolle, The Netherlands

**Keywords:** Absenteeism, Prediction models, Reclassification table, Risk distribution, Risk stratification, Sick leave

## Abstract

**Background:**

Prognostic models including age, self-rated health and prior sickness absence (SA) have been found to predict high (≥30) SA days and high (≥3) SA episodes during 1-year follow-up. More predictors of high SA are needed to improve these SA prognostic models. The purpose of this study was to investigate fatigue as new predictor in SA prognostic models by using risk reclassification methods and measures.

**Methods:**

This was a prospective cohort study with 1-year follow-up of 1,137 office workers. Fatigue was measured at baseline with the 20-item checklist individual strength and added to the existing SA prognostic models. SA days and episodes during 1-year follow-up were retrieved from an occupational health service register. The added value of fatigue was investigated with Net Reclassification Index (NRI) and integrated discrimination improvement (IDI) measures.

**Results:**

In total, 579 (51 %) office workers had complete data for analysis. Fatigue was prospectively associated with both high SA days and episodes. The NRI revealed that adding fatigue to the SA days model correctly reclassified workers with high SA days, but incorrectly reclassified workers without high SA days. The IDI indicated no improvement in risk discrimination by the SA days model. Both NRI and IDI showed that the prognostic model predicting high SA episodes did not improve when fatigue was added as predictor variable.

**Conclusion:**

In the present study, fatigue increased false-positive rates which may reduce the cost-effectiveness of interventions for preventing SA.

## Introduction

Sickness absence (SA) is an increasing problem in developed economies. The Organization for Economic Co-operation and Development reported that countries spend on average 2 % of their gross domestic product (GDP) and 10 % of their social expenditures on SA and disability benefits (OECD 2011). When off work due to sickness, the probability of resuming work decreases with increasing SA duration (Labriola 2008; Lund et al. 2008). Eventually, long-term SA leads to disability pension which excludes workers from the workplace and marginalizes them from the labor market. Therefore, it is important to identify high-risk workers before they report sick, so that they can be invited for interventions aimed at preventing SA (Taimela et al. 2008a; Kant et al. 2008).

Recently, two prognostic models for identifying workers at risk of high SA were developed in a sample of Dutch hospital workers (Roelen et al. 2013a) and validated in Dutch office workers (Roelen et al. 2013b) and Danish eldercare workers (Roelen et al. 2014a). The prognostic model predicting high SA days (i.e., ≥30 cumulated days during 1-year follow-up) showed fair performance in hospital workers, but poor performance at external validation. The prognostic model predicting high SA episodes (i.e., ≥3 episodes during 1-year follow-up) showed good performance in the development setting and maintained fair performance at external validation. It was concluded that more predictors of SA are needed to improve the SA prognostic models, particularly the model predicting high SA days.

Fatigue is a common symptom of ill-health, ranking third in prevalence after back pain and muscular aches in working populations (Parent-Thirion et al. 2012). Wikman et al. (2005) reported that fatigue was the most prevalent indicator of morbidity in the Swedish workforce. Several studies have already shown that fatigue is associated with future SA (Janssen et al. 2003; Bültmann et al. 2005, 2013; Åkerstedt et al. 2007; Roelen et al. 2014b). However, we need more research to investigate whether or not fatigue should be added as predictor to the SA prognostic models.

Traditionally, the added value of a new predictor to existing prognostic models is investigated by changes in the area under the receiver operating characteristic curve or *c*-statistic (Steyerberg et al. 2010). However, only very strong predictors can increase the performance of well-predicting prognostic models (Pepe et al. 2004; Janes et al. 2008; Cook 2007, 2008). The Net Reclassification Index (NRI) has been introduced as novel measure to assess the added value of predictor variables (Pencina et al. 2008; Steyerberg et al. 2010; Cook and Paynter 2011; Sundström et al. 2011). The NRI summarizes reclassifications of subjects when the new predictor is added. Subjects who develop the outcome are correctly reclassified when they move up into a higher risk category, and subjects who do not develop the outcome are correctly reclassified when they move down into a lower risk category. A disadvantage is that the NRI heavily depends on the thresholds of risk stratifications (Sundström et al. 2011). Pencina et al. (2011) defined an NRI without using risk categories, but this category-free NRI variant has been criticized for its high rates of false-positive conclusions (Pepe et al. 2014). The integrated discrimination improvement (IDI) is an alternative category-free measure to quantify risk discrimination improvement (Pencina et al. 2008; Steyerberg et al. 2010; Sundström et al. 2011).

Empirical evaluations of the literature showed that reclassification methods and measures are often applied inappropriately, for example, to assess the performance of new prognostic models that differ from the previously established models (Tzoulaki et al. 2011; Bouwmeester et al. 2012). In addition, the reclassification measures NRI and IDI are frequently misinterpreted (Kerr et al. 2014; Leening et al. 2014a). The objective of the present study was to introduce risk reclassification and related NRI and IDI measures in an occupational health context by investigating the added value of fatigue to the existing SA prognostic models. Reclassification analysis can be a key method for guiding decisions in occupational health care, because it presents the distribution of risks in the population and classifies subjects into relevant risk categories.

## Methods

To illustrate risk reclassification, we used the health check data from a previously described prospective cohort study of 1,137 office workers (Roelen et al. 2013b, 2014b). Predictor variables were measured by health check questionnaires administered in November 2006. SA in 2007 was retrieved from an occupational health service (OHS) register. The Medical Ethics Committee of the University Medical Center Groningen granted ethical clearance for the study.

### Baseline predictor variables

The health check questionnaire asked workers to rate their general health in categories ‘excellent’ = 4, ‘good’ = 3, ‘fair’ = 2, and ‘poor’ = 1 (Ware et al. 2002). Self-rated health (SRH) is widely used in health research and has been associated with various morbidity and mortality measures (Halford et al. 2012). The health check questionnaire measured fatigue with the checklist individual strength (CIS) consisting of 20 statements (Cronbach’s α = 0.92) on the severity of fatigue and its functional impact. The statements were scored on a seven-point scale ranging from 1 ‘fully agree’ to 7 ‘fully disagree’ amounting to a sum score ranging between 20 and 140, with higher scores reflecting higher levels of fatigue. The CIS was chosen because it has been validated for measuring fatigue in working populations (Beurskens et al. 2000; De Vries et al. 2003). Previous research has shown that the CIS has high internal consistency and good content validity for measuring fatigue (Beurskens et al. 2000; Dittner et al. 2004; Shahid et al. 2010). Convergent validity was satisfactory as reflected in correlations between the CIS and the Fatigue Assessment Scale (Pearson’s correlation coefficient *r* = 0.90; Michielsen et al. 2002), Fatigue Scale (*r* = 0.87; Chalder et al. 1993), and Maslach Burnout Inventory (*r* = 0.71; Maslach and Jackson 1986).

Age at baseline and SA in the 2 years prior to baseline were retrieved from the OHS register. SA was defined as temporary paid leave from work due to any (i.e., work-related and non-work-related) injury or illness and was recorded in the OHS register from the first SA day until return to work. The calendar days between the first and last SA day were counted as SA days, regardless of whether these were work days. SA days and episodes in the 2 years prior to baseline were accumulated for the predictor variables prior SA days and prior SA episodes, respectively (Roelen et al. 2013a, b). In agreement with Dutch SA insurance policies, SA episodes with less than 28 days worked between them were regarded as one episode.

### Outcome at 1-year follow-up

SA days and episodes in 2007 were retrieved from the OHS register. In previous SA prognostic studies, high SA days was defined as ≥30 cumulated (i.e., not necessarily consecutive) SA days and high SA episodes as ≥3 SA episodes during 1-year follow-up (Roelen et al. 2013a, b, 2014a). We adopted the same definitions for high SA days and episodes to ensure that the prognostic models in the present study did not differ from the established SA prognostic models.

### Statistical analysis

Descriptive statistics and logistic regression analysis were done with IBM SPSS Statistics for Windows, version 20.0 (IBM Corp. Armonk, NY, released 2011). Reclassification analyses were performed in R (Project for Statistical Computing) using the regression modeling strategies (rms) package (Harrell 2013) for calculating NRI and the predictABEL package (Kundu et al. 2011) for calculating IDI. Reclassification after adding fatigue was presented in reclassification tables (Cook 2008; Janes et al. 2008). It is recommended to distinguish between subjects with and without events in reclassification analysis (Pencina et al. 2008; Pepe 2011; Kerr et al. 2014; Leening et al. 2014a). Therefore, we summarized the reclassification of workers with high SA in the NRI for events (NRIe) and reclassification of workers without high SA in the NRI for nonevents (NRIne). NRIe = *P*(up|event) – *P*(down|event) representing the net proportion of subjects with events assigned to a higher risk category. NRIne = *P* (down|nonevent) – *P* (up|nonevent) representing the net proportion of subjects without events assigned to a lower risk category. The NRI varies between −100 and 100 %; positive values reflect improved classification, and negative values worsened classification.

Risk reclassification analysis requires calibrated models (Pepe and Janes 2011; Leening et al. 2014b). Calibration of the SA prognostic models with and without fatigue was addressed with the Hosmer–Lemeshow (H–L) goodness-of-fit test; adequate calibration was concluded for H–L *p* ≥ 0.05 (Steyerberg et al. 2010). After investigating the calibration of the SA prognostic models, we considered the models’ ability to stratify the population into risk categories. The risk categories should be clinically relevant in the sense that changing categories implies that subjects receive different treatments or interventions. As far as we know, relevant risk categories or risk thresholds have not yet been defined in SA research. Therefore, we chose two data-driven risk thresholds (10 and 20 % risk of high SA) based on previously reported risk distributions (Roelen et al. 2013b). In addition, we calculated the IDI to evaluate the improvement of the models’ ability to discriminate between high-risk and low-risk subjects without using categories. The IDI reflects the change in discrimination slope (i.e., difference between the mean estimated risk for cases and non-cases) of the model with the new predictor compared to the model with only the established predictors. IDI (range −100 to 100 %) represents overall risk discrimination improvement, but its magnitude is hard to interpret.

## Results

A total of 633 (56 %) office workers participated in the health checks. Non-participant analysis showed that participants were older (mean age = 44.5, standard deviation [SD] = 9.3 years) than non-participants (39.0, SD = 9.4 years; *t* test *p* < 0.01). Sixty-two percent of participants were men as compared to 68 % of non-participants (Chi-square *p* = 0.04). Fifteen percent of participants had high SA episodes compared to 22 % of non-participants (Chi-square *p* < 0.01). The proportions of office workers with high SA days did not differ (Chi-square *p* = 0.45) between participants and non-participants.

Fifty-four participants (8 %) had missing data on predictor and/or outcome variables, leaving 579 office workers with complete data for reclassification analysis (Table 1). Baseline fatigue was moderately correlated (Pearson *r* = 0.44; *p* < 0.01), but not collinear with SRH.

**Table 1:** Study population characteristics (*N* = 579)

Variable	Mean	SD	*N*	%
Age (years)	44.4	9.3		
Gender				
Men			361	62
Women			218	38
Work (h/week)	34.5	8.0		
Duration of employment (years)	3.2	1.3		
Self-rated health				
Excellent			143	25
Good			330	57
Fair			103	18
Poor			3	1
Prior sickness absence days				
0			118	20
1–7			143	25
8–14			104	18
15–30			94	16
31–60			60	10
>60			60	10
Prior sickness absence episodes				
0			118	20
1			130	22
2			115	20
3			74	13
4			64	11
>4			78	13
Fatigue (range 2–140)	51.2	21.0		

*SD* standard deviation

### Prognostic model for high SA days

Fifty-nine (10 %) office workers had high SA days during 1-year follow-up. Prior SA days, SRH and fatigue were significantly associated with high SA days, while age was not (Table 2). Calibration was adequate for the model without fatigue (H–L *χ* ^2^ = 13.8, *df* = 8; *p* = 0.09) and the model with fatigue (H–L χ^2^ = 4.7, *df* = 8; *p* = 0.79). The lower H–L model Chi-square indicated that the risks predicted by the prognostic model with fatigue were more in agreement with the observed frequencies of high SA days.

**Table 2:** Prospective associations with sickness absence (SA) days and episodes

Predictor	High (≥30) SA days			High (≥3) SA episodes		
	OR	95 % CI		OR	95 % CI	
Age	0.99^a^	0.74–1.32	*p* = 0.92	0.92^a^	0.70–1.21	*p* = 0.56
Prior SA	1.02^a^	1.00–1.08	*p* = 0.02	1.60	1.41–1.82	*p* < 0.01
Self-rated health	0.59	0.39–0.90	*p* = 0.01	0.56	0.37–0.84	*p* < 0.01
Fatigue	1.16^a^	1.03–1.31	*p* = 0.02	1.14^a^	1.01–1.28	*p* = 0.03

The table shows odds ratios (OR) and 95 % confidence intervals (CI) for univariate associations between predictor variables and sickness absence

^a^Per ten-point increase

At 10 % risk threshold, 84 workers (15 %) were reclassified when fatigue was added to the model. Seven workers with high SA days correctly moved up to the high-risk category, but four incorrectly moved down to the low-risk category. Table 3 shows that NRIe was 5.09 % and not significant (*p* = 0.91). Of the workers without high SA days, 73 were reclassified of whom 49 incorrectly. NRIne was −4.81 % reflecting significant (*p* < 0.01) worsening of the classification of workers without high SA days when fatigue was added (Table 3). At a more specific threshold risk of 20 %, NRIe was −1.69 % (*p* = 0.32) and NRIne was −1.15 % (*p* = 0.04). IDI was 0.25 % (95 % CI −0.25 to 0.75 %) indicating that fatigue did not significantly (*p* = 0.33) improve risk discrimination.

**Table 3:** Reclassification table for sickness absence (SA) days

Risk without fatigue	Risk with fatigue		Risk without fatigue	Risk with fatigue	
Events	≤10 %	>10 %	Events	≤20 %	>20 %
≤10 %	21	7	≤20 %	57	0
>10 %	4	27	>20 %	1	1
NRIe (95 % CI)	5.09 % (−5.93 to 16.10 %)		NRIe (95 % CI)	−1.69 % (−5.02 to 1.63 %)	
Nonevents	≤10 %	>10 %	Nonevents	≤20 %	>20 %
≤10 %	318	49	≤20 %	501	7
>10 %	24	129	>20 %	1	11
NRIne (95 % CI)	−4.81 % (−8.03 to −1.59 %)		NRIne (95 % CI)	−1.15 % (−2.22 to −0.09 %)	

*NRIe* Net Reclassification Index for events, *NRIne* Net Reclassification Index for nonevents, *CI* confidence interval

### Prognostic model for high SA episodes

Sixty-five (11 %) workers had high SA episodes at follow-up. Prior SA episodes, SRH and fatigue were significantly associated with high SA episodes, whereas age was not (Table 2). The prognostic models with fatigue (H–L χ^2^ = 3.0, *df* = 8; *p* = 0.94) and without fatigue (H–L χ^2^ = 2.1, *df* = 8; *p* = 0.98) were both well calibrated.

At a risk threshold of 10 %, seven workers (1 %) were reclassified when fatigue was added to the model. None of the workers with high SA episodes was reclassified (Table 4). Consequently, NRIe was not available. Of workers without high SA episodes, five were correctly reclassified as low risk and two were incorrectly reclassified as high risk; NRIne was 0.58 % and non-significant (*p* = 0.26). At a more specific 20 % risk threshold, four workers were reclassified after adding fatigue, although all incorrectly moved up to high risk. NRIne was −0.78 % and marginally significant (*p* = 0.05). IDI = 0.11 % (95 % CI −0.18 to 0.41 %) indicated that fatigue did not significantly (*p* = 0.45) improve risk discrimination.

**Table 4:** Reclassification table for sickness absence (SA) episodes

Risk without fatigue	Risk with fatigue		Risk without fatigue	Risk with fatigue	
Events	≤10 %	>10 %	Events	≤20 %	>20 %
≤10 %	16	0	≤20 %	33	0
>10 %	0	49	>20 %	0	32
NRIe (95 % CI)	n.a.		NRIe (95 % CI)	n.a.	
Nonevents	≤10 %	>10 %	Nonevents	≤20 %	>20 %
≤10 %	355	2	≤20 %	462	4
>10 %	5	152	>20 %	0	48
NRIne (95 % CI)	0.58 % (−0.43 to 1.59 %)		NRIne (95 % CI)	−0.78 % (−1.54 to −0.00 %)	

*NRIe* Net Reclassification Index for events, *NRIne* Net Reclassification Index for nonevents, *CI* confidence interval, *n.a.* not available

## Discussion

Fatigue is a core symptom of many medical conditions and an important indicator of morbidity in working populations. The present study confirmed that fatigue was prospectively associated with high sickness absence (SA) days and episodes during 1-year follow-up. However, reclassification analysis showed that fatigue did not improve risk predictions for office workers with high SA and worsened risk predictions for office workers without high SA.

Although reclassification analysis has become very popular, the NRI is often misinterpreted as ‘percentage of the population reclassified’ (Kerr et al. 2014; Leening et al. 2014a). In addition, the NRI equally values the consequences of false-negative and false-positive misclassifications. These problems were overcome in the current study by presenting NRI for events (NRIe) and nonevents (NRIne). Another problem is that data-driven risk thresholds may spuriously inflate the NRI (Sundström et al. 2011; Hilden and Gerds 2014). As there are no clinically relevant risk thresholds for SA, we dealt with this problem by analyzing reclassification at different (10 % and 20 %) risk thresholds. For the SA days model, NRIe was not significant, whereas NRIne was significant. For the SA episodes model, NRIne was on the verge of significance at the 20 % risk threshold.

However, the fact that NRIne was negative indicated worsened rather than improved risk classification of office workers without high SA. Workers without high SA days incorrectly moved up from the low-risk category to the high-risk category when fatigue was added as predictor variable. In other words, adding fatigue increased false-positive predictions of high SA. High false-positive rates can be problematic when resources are limited or when the burden and costs of interventions are high. Furthermore, Taimela et al. (2008b) reported that preventive consultations cost-effectively reduced SA only in high-risk workers and not in workers with a moderate or low SA risk. Hence, using fatigue as predictor not only increases unnecessary utilization of preventive interventions, but may also reduce cost-effectiveness.

Nowadays, there are many fatigue instruments because fatigue is recognized as a major symptom of clinical conditions. All instruments are self-report measures, and answers on self-report measures are driven by the respondent’s interpretation as well as factors such as personal dispositions, mood, expectations, and previous experiences. Furthermore, an instrument developed to measure fatigue in one patient group may not apply to other groups when fatigue experiences depend on the clinical condition. The CIS was previously shown to have good psychometric properties for measuring fatigue in working populations (Beurskens et al. 2000; Bültmann et al. 2002; De Vries et al. 2003). However, associations between fatigue and high SA days or episodes may differ when fatigue is measured with other instruments. Before drawing definite conclusions of the added value of fatigue to SA predictions, we need more studies investigating fatigue with different instruments or even a combination of instruments, because it is doubtful whether one instrument can capture fatigue, given the wide range of mechanisms underlying fatigue and the differing manifestations of fatigue (Dittner et al. 2004).

### Study strengths and limitations

The study population was limited to 1,137 office workers employed in one company, which is an advantage because it excludes SA variability due to differences in work conditions, work environment, and organizational policies and practices (Virtanen et al. 2008). The SA percentage (3.6 %) was slightly lower than in the Dutch workforce (4.2 %) in 2007, whereas the mean SA frequency in 2007 was 1.1 episodes in both the study population and the Dutch workforce (Statistics Netherlands 2014). However, this does not implicate that the results can be generalized to other working populations.

Another limitation of the study is that 56 % of the workers participated in the health checks. Janssen et al. (2003) reported a total CIS score of 53.4 in a heterogeneous sample (*N* = 7,495) of non-sicklisted workers participating in the Dutch Maastricht Cohort Study. The mean CIS score in the present study was 51.2 reflecting lower levels of fatigue (one sample *t* test *p* = 0.02). This might indicate that the office workers in our study were healthier. Such a ‘healthy volunteer effect’ (Etter and Perneger 1997; Froom et al. 1999) may have weakened associations between fatigue and high SA, consequently reducing the added value of fatigue to SA predictions.

## Conclusion

In the present study, fatigue increased false-positive rates which may reduce the cost-effectiveness of interventions aimed at preventing SA. The findings illustrate that we have to carefully consider the added value of potential predictors rather than strive for the most comprehensive prognostic models, even when we want to predict a complex multifactorial outcome such as SA. When applied and interpreted appropriately, risk reclassification can be used to gauge the added value of new predictors to established prognostic models.
